# Sequential Workflow for Integrated Biological Repair in Combined Four-Graft Knee Reconstruction

**DOI:** 10.1051/sicotj/2026044

**Published:** 2026-08-20

**Authors:** Eliseo Javier Firman

**Affiliations:** 1 Facultad de Ciencias Médicas, Pontificia Universidad Católica Argentina “Santa María de los Buenos Aires” (UCA) Buenos Aires Argentina; 2 Instituto Argentino de Diagnóstico y Tratamiento (IADT) Buenos Aires Argentina; 3 Grupo Médico Teuos Buenos Aires Argentina

**Keywords:** Osteochondral allograft transplantation, Meniscal allograft transplantation, Anterior cruciate ligament reconstruction, Cartilage restoration, Joint preservation, Complex knee reconstruction

## Abstract

*Objective:* To describe a structured technical-conceptual workflow for comprehensive biological knee reconstruction, focusing on the coordinated integration of four independent allografts – femoral osteochondral, tibial osteochondral, meniscal, and anterior cruciate ligament (ACL) – performed in a single operative setting. *Background:* Multicompartmental failure involving bipolar osteochondral defects, meniscal deficiency, and ligamentous instability represents one of the most challenging scenarios in joint preservation surgery. Although individual reconstructive techniques are well documented, current literature lacks a widely accepted consensus regarding their simultaneous execution. In this context, three-dimensional preoperative planning and structured sequencing logic are critical for preventing tunnel convergence and preserving the biological viability of the grafts. *Methodological Framework Description:* A structured reconstructive strategy is proposed to optimize surgical exposure and avoid spatial conflicts and graft interference. The workflow begins with a systematic arthroscopic mapping followed by a wide arthrotomy, adhering to a “surface restoration-to-stabilization” logic, consisting of: (1) customized femoral osteochondral shell allograft transplantation; (2) tibial osteochondral transplantation using a cylindrical plug technique; (3) medial meniscal allograft transplantation with dovetail bone fixation; and (4) anatomic ACL reconstruction using a soft tissue tendon allograft. This specific sequencing allows stabilizing elements to be adapted to the already restored articular anatomy, minimizing tunnel convergence and protecting soft-tissue grafts from aggressive osseous surgical maneuvers. *Clinical Relevance:* This technical-conceptual work provides a structured roadmap to support surgical decision-making in highly complex reconstructive cases. By systematizing operative sequencing and the management of technical interferences, the proposed workflow enables a reproducible biological reconstruction strategy aimed at restoring articular homeostasis in young and active patients within a single surgical procedure.

## Introduction

The coexistence of osteochondral defects, meniscal deficiency, and chronic anterior cruciate ligament (ACL) instability constitutes an extreme challenge for joint preservation. Since the functional balance of the knee depends on the synergistic interaction between the articular surface, the meniscus, and ligamentous stability, isolated repair of these components is often insufficient to restore joint homeostasis or halt degenerative progression [1]. This has driven the development of combined reconstructive strategies in young and active patients [2].

Both osteochondral allograft (OCA) and meniscal allograft transplantation (MAT) have demonstrated favorable clinical outcomes when utilized as standalone procedures [1]. However, surgical decision-making becomes critical when bipolar cartilage loss, meniscal deficiency, and ligamentous instability coexist. In this context, single-stage combined reconstruction allows for a comprehensive approach, avoiding the morbidity associated with staged procedures, although it introduces specific technical challenges related to intra-articular space management and tunnel convergence [1, 2].

In this study, the term “four-graft reconstruction” does not refer to a four-strand tendon graft classically used in ACL reconstruction [3], but rather to a combined reconstructive approach integrating four anatomically independent allografts – femoral osteochondral, tibial osteochondral, meniscal, and ligamentous – implanted sequentially in a single operative setting. Although this concept has been previously explored in complex scenarios [4, 5], its simultaneous execution lacks methodological standardization to resolve surgical sequencing conflicts and prevent graft interference.

The objective of this methodological review is to establish and describe a structured framework for combined biological knee reconstruction with four grafts. Emphasis is placed on a sequential workflow designed to mitigate technical challenges associated with tunnel interference and implant logistics, providing a roadmap for the safe and systematized execution of the procedure.

## Methods – surgical methodology framework

### Study design

This article represents a methodological and technical review aimed at presenting a reproducible surgical framework for single-stage multicomponent biological knee reconstruction. Its primary purpose is to analyze the preoperative planning, operative sequencing, and management of graft interference, rather than validating clinical outcomes or comparing isolated techniques.

### Indications and contraindications

Patient selection is critical for procedural reproducibility. Indications, contraindications, and selection criteria (alignment, functional demand, and patient adherence) are summarized in Table 1.

**Table 1:** Indications and contraindications. Concise summary including all original patient selection criteria.

Indications	Contraindications
Young active patients <50 years with high functional demand; selected patients 50–70 years with good alignment and preserved cartilage [2, 4, 5].	Advanced tricompartmental osteoarthritis, complete joint space loss, or diffuse collapse [1, 2, 4, 5].
Body mass index (BMI) <35 [4].	Significant arthrofibrosis with major range-of-motion limitation [2].
Symptomatic full-thickness bipolar osteochondral defects (ICRS III–IV) in medial or lateral femorotibial compartment [1, 4, 5].	Uncorrected major malalignment >3° varus or valgus without planned osteotomy [1, 4, 5].
Severe symptomatic meniscal deficiency after prior meniscectomy with persistent pain or overload [1, 2, 4, 5].	Untreated complex ligamentous instability compromises global knee kinematics [4, 5].
Chronic ACL rupture with clinically relevant rotational or anteroposterior instability [3].	Low functional demand or limited expectations of return to activity [4, 5].
Neutral or corrected mechanical alignment (±3°), or correctable during the same procedure (OCAT) [1, 4, 5].	Severe systemic comorbidities (peripheral vascular disease, uncontrolled diabetes, prior or active infection, synovial disease, collagenopathies) [2, 4, 5].
Absence of advanced osteoarthritis (Kellgren–Lawrence ≤ II) with preserved subchondral bone stock [1, 4, 5].	Inability to comply with rehabilitation protocol or expected poor adherence [2, 4, 5].
Prior indication for knee arthroplasty with partial preservation of joint space on weight-bearing flexion radiographs [2].	Untreated meniscal deficiency is a relative contraindication for cartilage restoration such as OCA [1].
Mandatory tobacco abstinence confirmed by urine testing [4].	Relative contraindications: active smoking, obesity, chronic corticosteroid use, or alcohol abuse [4, 6, 7].
High patient motivation and strict commitment to prolonged rehabilitation and postoperative restrictions [4, 5].	Mental health: reduced SF-12 mental component after complex reconstructions; requires psychological preparation and expectation management [5].

Proper patient selection is critical for procedural success. When one or more contraindications are present, alternative strategies such as staged treatment, palliative procedures, or arthroplasty should be considered [4, 5].

### Preoperative planning and methodological principles

Preoperative planning is the cornerstone of simultaneous multicomponent biological reconstruction, allowing for optimized graft selection and anticipation of technical difficulties [6, 7]. Clinical evaluation documents osteochondral, meniscal, and ligamentous involvement while ruling out contraindications.

Planning relies on a standardized imaging protocol [7], including weight-bearing radiographs, magnetic resonance imaging (MRI), and computed tomography (CT) for three-dimensional planning. In the presence of axial deformities or prior surgery, mechanical alignment and patellar height must be verified and, when necessary, corrected using techniques that preserve tibial slope and patellar height – such as biplanar “L”-shaped tibial osteotomy – thereby optimizing the biomechanical environment for complex biological reconstruction [8].

Precise quantification of bipolar defects and meniscal deficiency is mandatory. 3D planning enables the design of the femoral shell allograft and the selection of the tibial plug to achieve press-fit fixation and joint congruency [2]. Early communication with the tissue bank is essential to ensure graft compatibility and to verify quality and preservation criteria [6, 7].

### Sequential surgical workflow

The reconstruction is organized into a sequential workflow designed for the integrated implantation of all grafts in a single setting. The sequence is structured into four consecutive stages – femoral shell, tibial plug, meniscal dovetail, and anatomic ACL – to facilitate recipient bed preparation and reduce the risk of technical interference. To further illustrate this methodology, a step-by-step surgical video of the complete sequential workflow is provided (Supplementary Video 1).

The distinguishing features of this approach and its technical advantages are summarized in Table 2.

**Table 2:** Key technical highlights Summary of the main technical features of the single-stage sequential biological reconstruction.

Technical feature	Associated technical advantage
Simultaneous reconstruction of osteochondral, meniscal, and ligamentous structures in a single surgical stage.	Allows comprehensive knee restoration while avoiding staged procedures, with a single rehabilitation period and reduced overall recovery time.
Ordered four-step sequence (femoral shell, tibial plug, meniscal dovetail, and anatomic ACL reconstruction).	Enhances procedural reproducibility, optimizes tunnel orientation, and reduces the risk of graft interference.
Use of a dovetail meniscal fixation technique.	Provides stable bone fixation, improves physiological load transmission, and promotes biological integration of the graft.
Adaptability of the technique to different graft combinations.	Allows customization according to allograft availability and surgeon preference without altering the core reconstructive concept.
Three-dimensional preoperative planning and simultaneous preparation of recipient beds.	Reduces the risk of tunnel convergence, optimizes final articular congruency, and improves reconstructive accuracy.

### Patient positioning

Surgery is performed under combined regional and general anesthesia, with the patient in the supine position on a radiolucent table and the affected limb placed in a mobile leg holder, allowing a full range of flexion and extension. Regional anesthesia consists of an ultrasound-guided posterior popliteal sciatic block combined with general anesthesia to optimize perioperative analgesia and anesthetic management during this prolonged multistage procedure. A proximal pneumatic tourniquet is applied at 300 mmHg, and the surgical field is prepared from the proximal thigh to the distal third of the leg. In experienced hands, the complete sequential procedure typically requires approximately 4 h of operative time, although this may be prolonged during the learning curve depending on graft complexity and intraoperative technical demands. The tourniquet is generally maintained during the initial stages of the procedure and subsequently managed in a staged manner according to the requirements of each reconstructive phase, with selective release or intermittent deflation when necessary, balancing surgical field control with tissue perfusion and avoiding unnecessarily prolonged continuous ischemia. Standard intraoperative anesthetic monitoring and fluid management are maintained throughout the procedure due to the prolonged operative time and the multistage reconstructive nature of the surgery.

### Initial arthroscopic assessment and surgical approach

The procedure begins with a systematic diagnostic arthroscopy to confirm the indication for combined reconstruction. The magnitude of the defects is determined, and lesions are classified according to the ICRS scale [9, 10]. Selective synovectomy, chondroplasty, and margin preparation are performed without initiating the reconstructive phase [10, 11]. If necessary, maneuvers to optimize access, such as notchplasty or medial collateral ligament *pie-crusting*, are employed [12].

Following the arthroscopic assessment, an extended medial parapatellar approach is performed, allowing simultaneous access to the compartments for sequential preparation and implantation. This hybrid approach combines initial arthroscopic precision with the open exposure required for the reconstructive phase.

Osteochondral transplantation may be performed using a plug technique for small focal defects or a shell graft technique for large or irregular defects, with comparable integration and survivorship when appropriate indication and articular congruence are respected [7].

Because chondrocyte viability is a key determinant of OCA transplantation and closely related to graft preservation, storage conditions, and time from procurement to implantation, graft suitability is based on tissue-bank release criteria and quality-control documentation before surgical use [7]. Direct intraoperative cellular viability testing is not routinely performed in this workflow. During surgery, osteochondral grafts are prepared and implanted sequentially, minimizing unnecessary back-table exposure, desiccation, and prolonged manipulation before implantation. Rather than applying a rigid intraoperative time cut-off, priority is given to efficient sequential graft handling and minimization of unnecessary graft exposure once preparation has been completed.

### Surgical exposure

**Figure 1:**
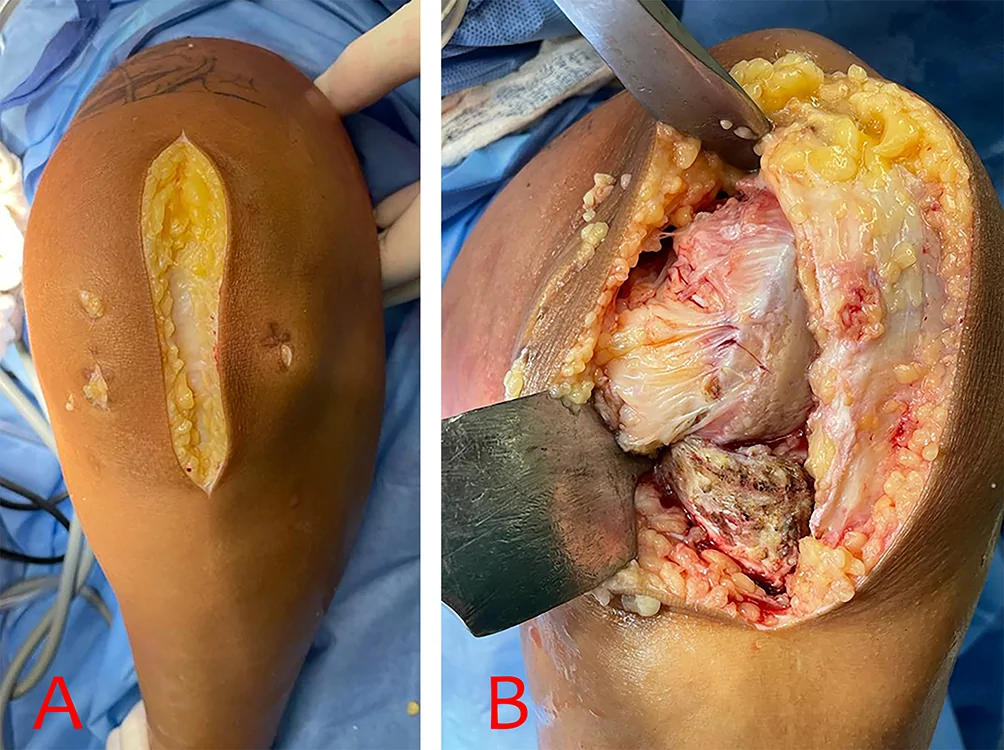
(A) Anterior longitudinal approach. (B) Joint exposure with Hohmann retractors placed in both compartments.

The anterior longitudinal incision extends from 4 cm proximal to the patella to 1 cm distal to the tibial tuberosity. After the arthrotomy, the proximal tibial metaphysis is exposed by subperiosteal elevation of the medial capsular flap and the deep medial collateral ligament, with controlled patellar eversion. The strategic placement of Hohmann retractors ensures adequate visualization throughout the workflow (Figure 1).

## Step 1 – Femoral condyle osteochondral shell allografting

The osteochondral shell graft technique enables the reconstruction of extensive or irregular defects using osteochondral allografts (OCA) without donor-site morbidity [7, 10]. Within this sequential workflow, restoring the femoral condyle is the primary step to establish the articular surface reference frame. As this technique does not rely on proprietary instrumentation, it demands high manual precision to ensure joint congruency [7].

### Recipient bed preparation

The femoral condyle defect is delimited and regularized into a geometric shape (rectangular or trapezoidal) to optimize graft seating. After defining the borders with a scalpel, tangential reaming is performed until healthy, bleeding subchondral bone is exposed, maintaining a uniform depth of 4–5 mm. It is critical to preserve the anatomic radius of curvature of the recipient condyle to prevent overstuffing or articular incongruence. Final dimensions are transferred to the allograft using a sterile template [7].

### Graft preparation

The graft is harvested under sterile conditions using an oscillating saw, maintaining a slight initial oversizing to allow for high-precision progressive adjustment. The final thickness of the shell graft should be 6–7 mm [4]. Subchondral microperforations are performed, followed by pulsatile lavage with 1.5 L of saline solution to remove immunogenic marrow elements. Optionally, the graft may be impregnated with bone marrow aspirate concentrate (BMAC) to enhance biological integration [4, 7].

### Graft implantation

The graft is implanted using a press-fit technique, ensuring intimate cartilage-to-cartilage and bone-to-bone contact [7]. Progressive adjustments are made with a sagittal saw and burr until an exact and stable fit is achieved. It is essential to verify articular congruency throughout the full range of motion, avoiding steps, incongruities, or overstuffing that could alter joint biomechanics [7].

**Figure 2:**
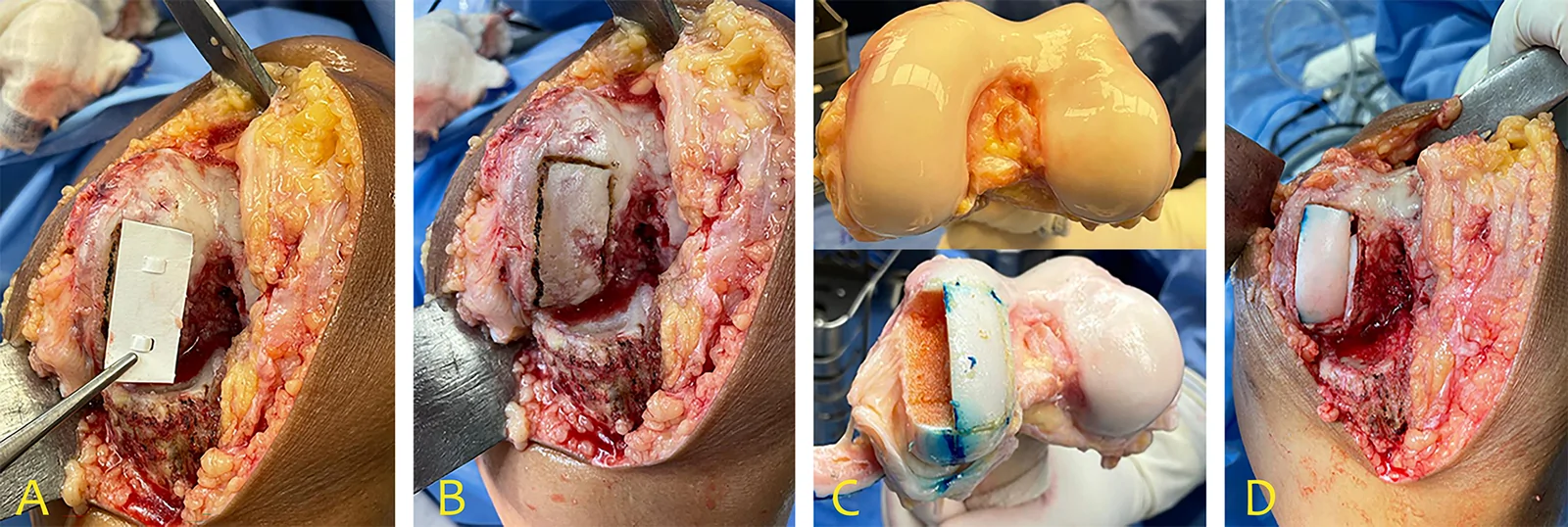
(A, B) Preparation of the recipient bed: the osteochondral defect is outlined and sized using a sterile template. (C) Shaping of the customized shell-type graft (tab-in-slot configuration). (D) Final result after implantation of the femoral osteochondral block graft (shell technique) with complete anatomic adaptation to the recipient bed.

No additional fixation systems are used, as the press-fit provides sufficient primary stability to allow for progressive biological integration of the graft (Figure 2).

## Step 2 – Tibial osteochondral transplantation (press-fit plug technique)

At this stage, the use of OCA is preferred over autograft (OAT) to avoid donor-site morbidity and to enable the reconstruction of larger bipolar defects with viable hyaline cartilage [7].

### Recipient bed preparation

The bipolar osteochondral defect in the medial tibial plateau is identified, and controlled cylindrical reaming is performed using calibrated trephines and specific guides. The recipient bed is prepared to a depth of 6–10 mm until healthy, bleeding subchondral bone is reached, preserving the peripheral margins to maintain stability and congruency [7]. Reaming must be performed perpendicular to the articular surface, avoiding over-resection and protecting the integrity of the posterior tibial cortex.

### Graft preparation

The cylinder is harvested with a diameter identical to the recipient bed to ensure a press-fit adjustment. The bone edges are gently beveled to facilitate insertion [7].

In the case of allografts, the cylinder is obtained from a donor site selected according to the closest possible contour match and anatomic orientation – typically the donor’s femoral condyle – allowing for the maintenance of joint congruency and hyaline cartilage continuity, even if the graft source differs anatomically from the recipient site.

### Curvature mismatch and tibial graft selection

Although tibial-to-tibial osteochondral matching theoretically represents the preferred anatomical option, as it may better reproduce the native radius of curvature and overall contour congruity of the recipient surface [13], simultaneous availability of multiple size-matched tibial allografts may be challenging in complex combined reconstructions involving simultaneous tibial osteochondral and meniscal restoration [4].

In these scenarios, osteochondral plugs harvested from the donor femoral condyle may represent a pragmatic alternative. However, the convex morphology of the femoral condyle differs from the relatively flat or slightly concave medial tibial plateau, creating a potential risk of geometric incongruity, edge loading, and focal subchondral overload, factors associated with potential graft failure in OCA transplantation [4, 13].

To minimize this limitation, graft selection is based not only on diameter matching but also on macroscopic surface congruity, cartilage contour compatibility, and anatomic orientation. Following the technical principles described by Wang and Bugbee [13], the recipient site is reamed perpendicular to the articular surface, and defect depth is measured at the 12-, 3-, 6-, and 9-o’clock positions to reproduce the corresponding graft geometry. The graft is subsequently contoured according to these measurements, and the edges are gently beveled to facilitate congruent insertion and minimize graft prominence or peripheral step-off [13]. Likewise, the donor area is selected according to the closest possible radius of curvature and contour match relative to the recipient surface [13].

Final implantation requires careful dynamic intraoperative assessment throughout the full range of motion to verify surface congruency and avoid peripheral step-off or focal overload [13].

Biomechanical evidence specifically validating femoral-to-tibial osteochondral matching remains limited, and this strategy therefore should not be considered an ideal anatomical reconstruction model. Accordingly, this approach differs from the standard anatomical matching principles generally described for conventional OCA transplantation [13]. Instead, it should be interpreted as a technically pragmatic solution reserved for highly selected complex reconstructive scenarios with tissue-bank logistical constraints and the need for simultaneous biological restoration of multiple structures.

If OAT is selected, the cylinder is extracted from a non-weight-bearing area of the femur, respecting the depth of the defect. The osteochondral architecture is preserved to allow for anatomical restoration with viable hyaline cartilage and subchondral bone [10].

### Graft implantation

The cylinder is transplanted while ensuring the integrity of the tidemark, as its continuity is crucial for biomechanical integration [10]. The graft is introduced via manual pressure or controlled impaction until it is flush or slightly recessed relative to the surrounding surface, achieving stable primary fixation without the need for screws. Subsequently, surface congruity is verified throughout the full range of motion to rule out any steps or overstuffing.

**Figure 3:**
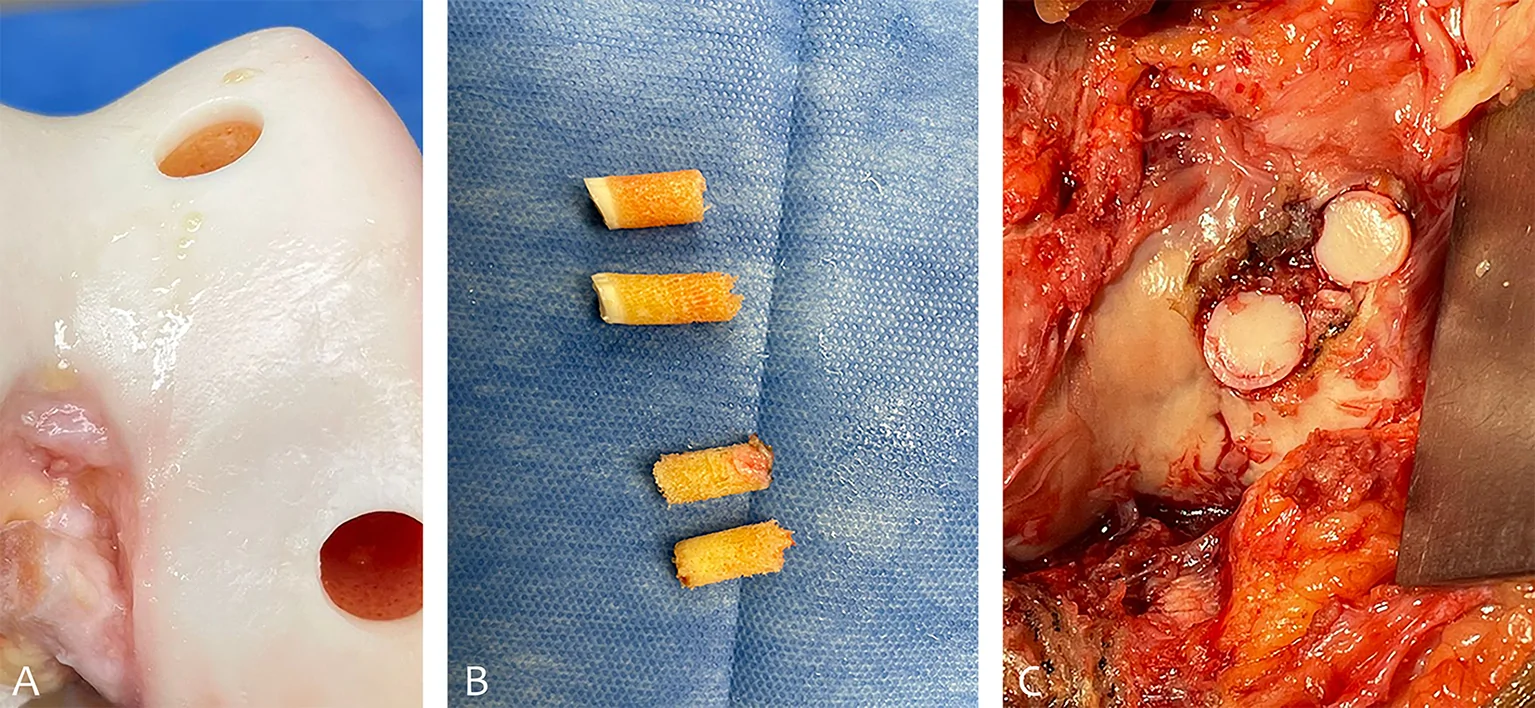
Tibial osteochondral transplantation using the plug technique. (A) Harvesting of the osteochondral cylinder from the femoral condyle of the cadaveric donor. (B) Osteochondral allograft cylinders prepared with calibrated trephines. (C) Osteochondral graft implanted into the medial tibial plateau defect using the press-fit technique.

For wide, oval, or irregular defects exceeding the diameter of a single plug, a “snowman” technique may be employed using adjacent or overlapping cylinders [7]. Depending on the extent of the lesion, either a single cylinder or a multi-graft configuration (mosaicplasty) is used. The interstitial spaces between the cylinders typically fill spontaneously with fibrocartilage during the biological healing process [10] (Figure 3).

### Biological advantages and additional considerations

The primary advantages of this anatomical and biological technique include the use of viable hyaline cartilage, the preservation of joint congruency and height, and direct osseous consolidation between the graft and the recipient bone. Although peripheral cartilage integration may be incomplete, functional tissue continuity and adequate load distribution are maintained [10].

The first two steps of this bipolar reconstruction (femur and tibia) can be complemented with additional transplants – such as patellar, trochlear, or opposite compartment OCA – in cases of extensive focal grade III–IV chondral defects [4, 10]. This versatility allows the methodological framework to be adapted to multicompartmental lesions without altering the sequential logic of the procedure.

## Step 3 – Medial meniscal allograft transplantation (dovetail technique)

Medial meniscal allograft transplantation (MAT) is performed as part of a comprehensive knee reconstruction to protect the restored articular surface and mitigate the risk of failure [2, 11, 12]. Graft sizing is determined preoperatively via plain radiographs using the Pollard method [12]. In this workflow, a meniscal graft attached to a dovetail bone bridge is used, allowing for simultaneous fixation of both horns while preserving their anatomic relationship within a single tibial slot. This configuration may facilitate anatomic horn positioning and simplified management of the tibial bone stock [11].

Although dovetail bone-bridge fixation has been primarily described for lateral meniscal allograft transplantation, specific evidence validating dovetail fixation for medial MAT remains limited. Its use in the present medial reconstruction was adapted to address the coexistence of the tibial osteochondral plug, the meniscal bone block, and the tibial ACL tunnel within the same proximal tibial construct. Unlike the lateral meniscus, the medial meniscus presents a greater distance between the anterior and posterior roots, a more “C-shaped” configuration, and a different anatomical relationship with the tibial spine and ACL insertion [11].

### Recipient bed preparation

The meniscal remnant is debrided, preserving a stable 1–2 mm peripheral rim as a capsular buttress. In cases of total meniscal absence, extreme care is taken to prevent extrusion [12]. A medial tibial recipient slot with dovetail geometry is created, preserving the peripheral cortex and the meniscotibial ligament. Slot preparation is performed according to the geometry of the selected meniscal allograft bone bridge, aiming to reproduce a compatible trapezoidal recipient slot and allow accurate graft seating within the tibial trough [11]. In contrast to the more compact lateral configuration, the medial slot is oriented more longitudinally and peripherally to reproduce the wider anteroposterior root separation and more C-shaped geometry of the medial meniscus while preserving adequate bone stock between the slot, the tibial osteochondral recipient bed, and the planned ACL tibial tunnel. The anterior length of the slot is initially left longer to be adjusted according to the final tibial depth. The location of this slot is coordinated with the osteochondral transplant from Step 2 to ensure the structural integrity of the tibial plateau.

### Graft preparation

The allograft is prepared on a sterile back-table simultaneously. The meniscal bone bridge is contoured to reproduce the trapezoidal geometry of the recipient slot, with a lateral oblique and medial vertical cut. Slight intraoperative adjustments may be required to ensure accurate fit and smooth insertion within the tibial trough [11]. The horns are identified, and traction sutures are placed for intraoperative graft control. The posterior bone block is trimmed flush with the posterior horn, while the anterior portion is initially left oversized to allow for definitive tensioning during implantation [11]. Special attention is directed toward maintaining anatomic horn orientation and avoiding graft prominence within the tibial trough, as medial meniscal extrusion may be favored by mismatch, overconstraint, or nonanatomic positioning, particularly given the greater excursion of the medial meniscus relative to the lateral compartment.

### Implantation and fixation

**Figure 4:**
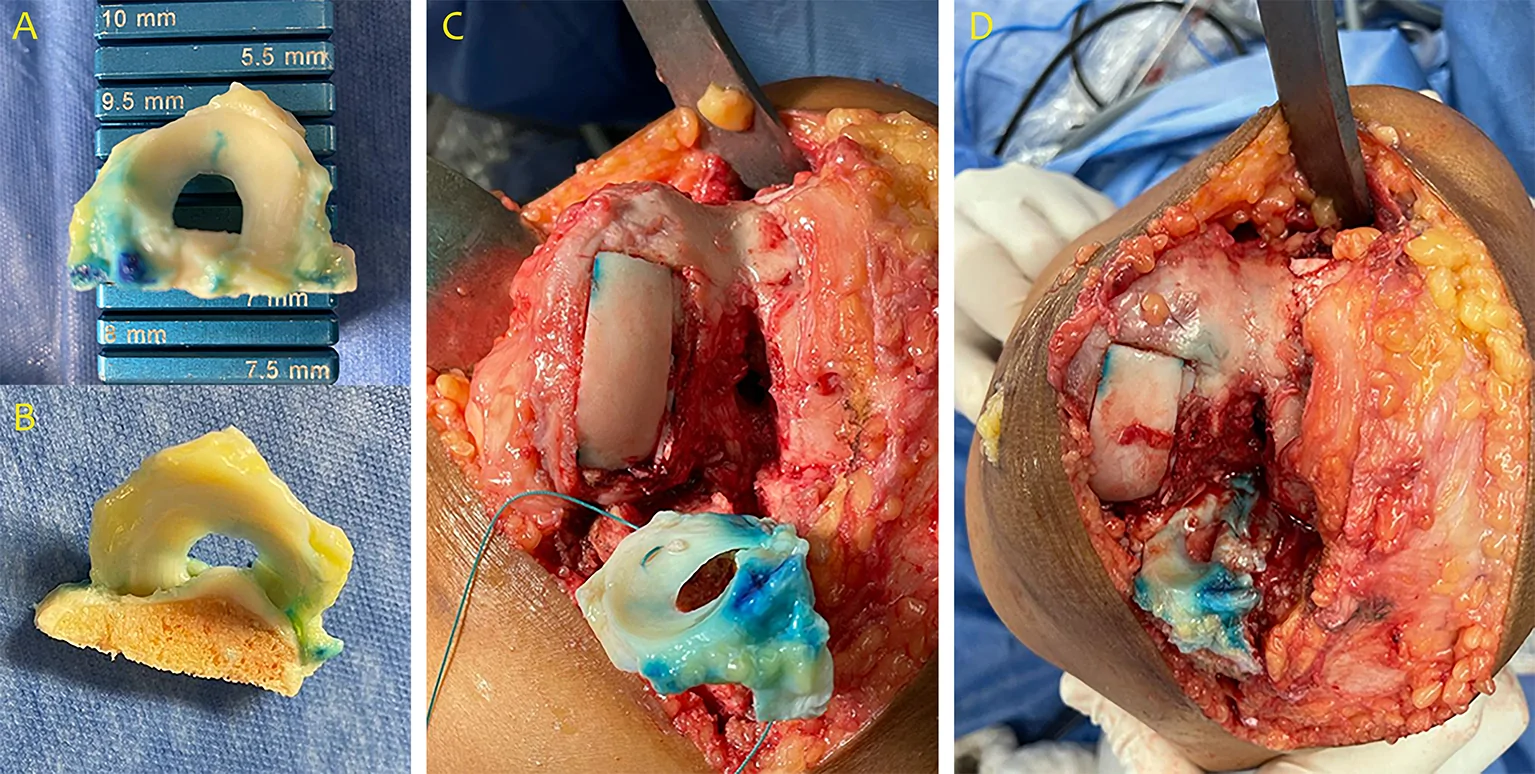
Medial meniscal allograft transplantation – Dovetail technique. (A, B) Meniscal graft attached to a rectangular, dovetail-shaped bone block. (C) Positioning of the graft on the cutting station, aligning the trapezoidal contour of the bone block with the fixation posts. (D) Stable press-fit insertion of the bone block into the tibial slot with complementary peripheral fixation using a bucket-handle suture configuration.

The bone block is inserted into the slot using a press-fit technique until an exact fit is achieved. Peripheral fixation is performed from posterior to anterior using a combination of *all-inside, inside-out,* and *outside-in* techniques (8–10 sutures). Fixation of the anterior horn is reserved for the final stage, allowing for fine adjustments in length and tension. These sutures are organized and maintained but not definitively tied until the implantation of the osteochondral and ligamentous grafts is complete, preventing premature tension that could displace the graft [11, 12]. This sequential fixation strategy also allows dynamic reassessment of meniscal position and peripheral tension before final fixation, aiming to minimize extrusion and avoid excessive constraint during the final stages of reconstruction (Figure 4).

## Step 4 – Anterior cruciate ligament (ACL) reconstruction

Anatomical ACL reconstruction represents the final stage of the procedure. In the context of this combined reconstruction, rigorous spatial planning is essential to prevent mechanical interference with the osteochondral grafts and the meniscal bone bridge.

### Graft selection

In the present four-graft biological reconstruction framework, an all-allograft strategy is intentionally prioritized. Although autografts remain valid options in isolated ACL reconstruction, their use in this specific multicomponent setting may add donor-site morbidity, increase surgical aggression, and introduce additional tissue-harvest demands during an already complex reconstructive procedure. For this reason, autografts are not preferred in the proposed workflow.

Available allograft options for ACL reconstruction include soft-tissue grafts (such as anterior or posterior tibialis tendon allografts) and bone-block grafts (such as BPTB or Achilles tendon allografts) [12, 14, 15]. Recent comparative evidence has shown similar clinical outcomes, functional results, complication rates, and failure rates among these commonly used allograft types in primary ACL reconstruction [14]. In the present workflow, anterior tibialis tendon allograft was selected as the preferred soft-tissue graft for this specific multicomponent reconstructive setting. Furthermore, anterior tibialis tendon allograft is preferred because its soft-tissue configuration avoids the incorporation of additional bone blocks within a proximal tibia already occupied by the tibial OCA bed and the meniscal dovetail bone bridge, thereby reducing the risk of spatial interference while maintaining a biological allograft-based reconstructive strategy [14].

### Preparation and tunneling

Anterior cruciate ligament (ACL) remnants are resected while preserving the femoral and tibial footprints as fundamental anatomical landmarks [15, 16].

Femoral Tunnel: Performed using an independent anteromedial technique with the knee at 120° of flexion. Under direct visualization (facilitated by the previous arthrotomy), this technique allows for a tunnel orientation that minimizes the risk of convergence with the femoral osteochondral graft [15, 16]. The center of the tunnel is localized at the native anteromedial (AM) footprint, between the bifurcate ridge and the inferior margin of the notch, 5 mm from the posterior border and 5 mm from the inferior margin [17]. Typically, a 5 mm cortical bridge is drilled for the button, followed by a 10 mm socket matching the graft diameter.

Tibial Tunnel: Positioning is critical and must be coordinated with the dovetail meniscal slot and the edge of the tibial OCA. The tunnel is reamed to the graft diameter, aiming for an orientation of 60°–65° in the coronal plane (relative to the medial joint line) and 50°–55° in the sagittal plane, to preserve bone stock and avoid spatial conflict with the meniscal transplant. These angulation parameters serve as practical initial references based on available ACL and combined-reconstruction literature [15–17], and are intended as starting intraoperative guides rather than rigid geometric rules for the present four-graft construct. Rather than representing a fixed geometric rule, final tunnel orientation is dynamically adapted intraoperatively according to the residual bone stock, the previously established graft constraints, and direct surgical assessment of the proximal tibial plateau.

In the present workflow, tibial tunnel drilling is intentionally performed only after completion of the tibial osteochondral plug and meniscal dovetail fixation, allowing the final ACL tunnel trajectory to be adapted to the already established osseous constraints rather than forcing previous grafts to accommodate a predefined ligament tunnel. This sequential logic prioritizes preservation of residual proximal tibial bone stock and permits real-time adjustment of tunnel trajectory according to the effective spatial constraints created by the previously fixed grafts.

Fluoroscopic control may be used as an adjunct in selected cases to confirm final tunnel orientation and hardware position; however, this assistance does not replace direct surgical planning or eliminate the need for careful intraoperative judgment.

### Placement and fixation

A combination of suspensory femoral fixation and tibial interference fixation is utilized. The graft is implanted using a single-bundle anatomical technique with a self-adjusting suspensory device in the femur.

**Figure 5:**
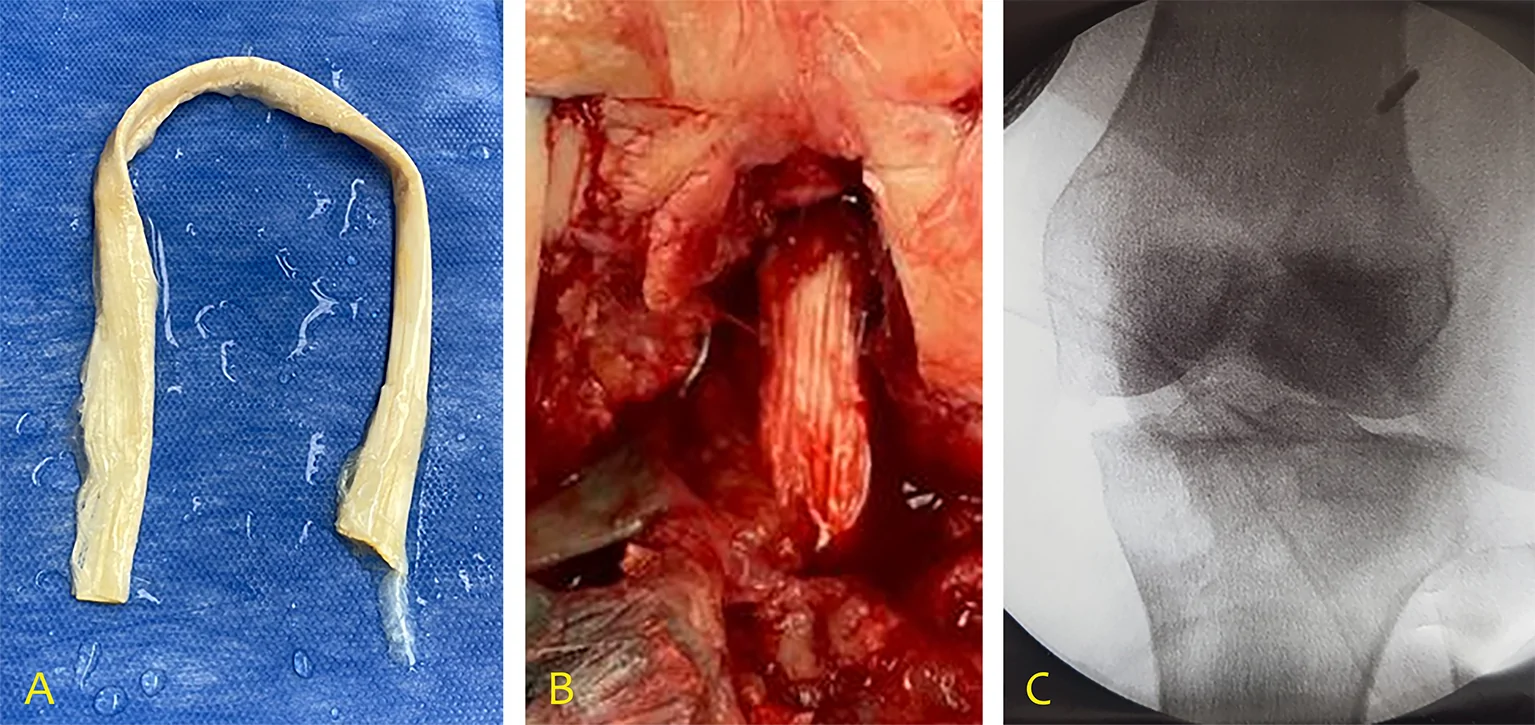
Anterior cruciate ligament reconstruction. (A) Anterior tibialis tendon allograft prepared for implantation. (B) Intra-articular positioning of the graft and final fixation. (C) Fluoroscopic control showing anatomic tunnel placement and femoral cortical fixation of the graft.

Once the ACL is positioned, the knots of the meniscal sutures (Step 3) are completed, and the knee is cycled 20 times under tension to minimize the creep effect. After confirming the absence of impingement, tibial fixation is performed in full extension using an interference screw 1 mm larger than the tunnel diameter. Finally, global stability and biomechanical congruency are verified before closure. In selected cases, a supplemental tibial staple may be added [18] (Figure 5).

## Tips and pearls vs pitfalls – technical recommendations

The tips, pearls, and pitfalls for single-stage four-graft knee reconstruction are summarized in Table 3.

**Table 3:** Tips and pearls vs pitfalls – technical recommendations.

Tips and pearls	Pitfalls and prevention
Precise patient selection with neutral alignment and high rehabilitation adherence.	Incorrect indications in advanced OA or uncorrected deformities lead to early failure.
Accurate 3D preoperative measurements of cartilage, tibial plateau, and meniscal size.	Graft–host incongruity causing step-offs, overload, and degeneration.
Early coordination with tissue bank to secure fresh-frozen, non-irradiated grafts.	Convergence of tibial tunnels with dovetail slot or OCA beds.
Systematic diagnostic arthroscopy before open reconstruction.	Meniscal extrusion due to oversized grafts or premature anterior fixation.
Selective MCL pie-crusting to improve visualization and working space.	Femoral shell graft overstuffing causes postoperative pain.
Posterior horn traction sutures were placed before meniscal insertion.	Neurovascular injury during posteromedial meniscal repair.
Routine use of a knot pusher for secure meniscocapsular fixation.	Improper allograft handling reduces cellular viability.
Continuous intraoperative meniscal centering and balancing.	Incorrect ACL graft tensioning causes abnormal knee kinematics.
Precise femoral shell graft shaping to restore native condylar curvature.	Accelerated rehabilitation leading to graft loosening or extrusion.
Pre-placed vertical sutures for uniform peripheral meniscal fixation.	Rare vascular complications: Deep posteromedial dissection and osseous tunnel creation carry a risk of popliteal branch injury. Blind maneuvers must be strictly avoided. A severe arterial injury with pseudoaneurysm, hypovolemic hemorrhage, compartment syndrome, and need for endovascular embolization and fasciotomy has been previously reported by the authors during hamstring harvest [19]. This underscores the importance of meticulous dissection and early vascular assessment when disproportionate postoperative pain or swelling is observed.
Dynamic ROM verification after each reconstructive step.	
Delayed final meniscal knot tying until cartilage and ACL reconstruction are completed.	
Sequential order: femoral OCA → tibial OCA → meniscus → ACL.	

### Postoperative rehabilitation protocol

Follow-up visits are scheduled at 2 weeks, 6 weeks, 3 months, 6 months, 1 year, and annually thereafter. Standardized radiographs are obtained at each visit, with the exception of the initial 2-week follow-up. Rehabilitation is structured to preserve range of motion while minimizing mechanical overload, with progression primarily dictated by the biologically limiting graft, particularly osteochondral allograft integration, while also respecting meniscal healing and ligament graft maturation [4, 7, 12, 15]. The postoperative rehabilitation protocol is summarized in Table 4.

**Table 4:** Postoperative rehabilitation protocol.

Phase	Timeframe	Main goals	Weight bearing	Range of motion	Key exercises/activities
Phase I – protection and early mobilization	0–6 weeks	Graft protection, edema control, quadriceps activation	Non–weight-bearing 0–4 weeks; partial up to 50% at 4–6 weeks	0°–90° (first 4 weeks)	Hinged brace locked in extension; isometric quadriceps; patellar mobilization; CPM or assisted ROM; cryotherapy and limb elevation
Phase II – recovery of motion and transition to full weight bearing	6–12 weeks	Restore ROM, initiate strength, gait normalization	Progressive advancement toward full weight bearing from week 6, according to biological graft integration	Gradual recovery to full ROM by weeks 10–12	Closed-chain strengthening; basic proprioception; neuromuscular re-education; ADLs allowed at ~12 weeks
Phase III – strengthening and functional retraining	3–6 months	Strength, dynamic control, neuromuscular coordination	Full	Full	Progressive resistance training; advanced proprioception; return to low-demand work if clinically appropriate
Phase IV – progressive functional return	6–18 + months	Functional performance, impact control	Full	Full	Cycling, elliptical, swimming (6 months); linear jogging at 9 months if asymptomatic; pivoting/impact sports ≥18 months

The timelines presented below should be interpreted as biological and functional reference milestones rather than rigid chronological rules.

### Special considerations

- Criteria-based progression: Rehabilitation advancement must be guided by objective clinical, functional, and biological criteria rather than chronological timelines alone. Persistent pain, synovitis, effusion, or signs of instability require re-evaluation before progression to the next phase.
- Biological integration: In combined OCA + MAT + ACL reconstruction, progression is primarily determined by the biologically limiting graft – particularly osteochondral allograft integration – while respecting meniscal healing and ligament graft maturation. Premature loading or return to sports may increase the risk of graft failure.
- Impact activities: In the setting of multiple biological reconstructions (OCA + MAT + ACL), impact sports should generally be considered contraindicated. Any exception should be based on individualized assessment and cautious progression.

## Discussion

The coexistence of extensive osteochondral defects, meniscal deficiency, and ligamentous instability creates an adverse biomechanical environment where isolated treatment rarely restores joint function or halts degenerative progression [1, 2]. Consequently, joint preservation strategies in young, active patients require integrated approaches that simultaneously address osteochondral congruence, meniscal load transmission, and ligamentous stability.

The primary contribution of this work lies not in the description of each individual reconstructive procedure – all of which are widely reported in the literature – but in the sequential integration methodology required to combine them safely and reproducibly in a single-stage surgery. The proposed workflow begins with a systematic arthroscopic review; this step is crucial for definitive mapping of the lesions under fluid distension and confirming the reconstructive indication, as well as ruling out concomitant pathology in unaffected compartments that could compromise the prognosis of the biological reconstruction. Subsequently, a wide arthrotomy is performed. This approach should not be interpreted merely as a surgical access route, but as a fundamental strategic decision: direct three-dimensional visualization eliminates the optical distortions of arthroscopy and ensures the precise navigation required for subsequent steps [15].

Following this sequential logic, the reconstruction begins with the femoral shell graft (Step 1) and the tibial plug cylinder (Step 2). These initial steps serve as the reference pillars for the biological reconstruction; restoring the weight-bearing bony surfaces first allows for the definition of joint height and congruence before proceeding with the meniscal block and the ACL. This ensures that the available surgical space is not compromised by errors in the impaction of the osteochondral grafts [7, 20]. This strategy is notable for its methodological adaptability, as it is independent of the specific type of graft used (fresh, cryopreserved, irradiated, or alternative structural grafts), according to local availability and surgeon preference, without altering the sequential logic of the procedure [21, 22].

Osteochondral reconstruction using plug or shell grafts has shown satisfactory results when indication and joint congruence are respected [7, 10, 13, 20]. Complementarily, meniscal allograft transplantation (MAT) restores physiological load transmission, while anatomical ACL reconstruction is fundamental to reestablish stability and reduce secondary overloading on the grafts [1, 2, 4, 14, 20, 23]. While these procedures offer partial benefits in isolation, their sequential methodological integration addresses the pathology in a global and physiological manner.

### Technical rationale: Choice of meniscal fixation method

The fixation technique for MAT is a determining factor in the success of multicomponent reconstructions. Although the use of bone plugs with independent suspensory fixation allows for individual tensioning of each horn, its execution in a bipolar reconstruction (following a tibial OCA) significantly increases the risk of bone fracture, tunnel convergence, and collision with the osteochondral graft [4, 12]. Evidence demonstrates that bone fixation techniques better reproduce native biomechanics, particularly the keyhole/bone bridge technique, which achieves a load distribution similar to the intact meniscus [11, 12].

Multiple bone bridge variants have been described, such as the trough, keyhole, and bridge-in-slot [11]. However, some require more aggressive central bone resection, potentially compromising the stability of the underlying tibial OCA or interfering with the ACL tunnel trajectory. In contrast, the dovetail technique offers proven biomechanical advantages in stability and reduced micromotion, thereby optimally preserving the anatomical distance between the meniscal horns (2.5–3 cm) [11, 12]. From a methodological perspective, the use of a single dovetail bone block is justified because it facilitates simultaneous anatomical fixation of both horns, physiological load transmission, and simplified bone-to-bone integration through a single tibial slot with a press-fit adjustment [11]. As limitations, this technique demands high geometric precision to avoid extrusion or overstuffing and does not allow for independent tensioning of each horn. Sizing must be exact, as undersized grafts are associated with compartmental overload, while oversized grafts show a greater tendency toward extrusion. Furthermore, the slot location must be planned to avoid including the meniscus within the tibial OCA, preventing critical fixation interferences.

### Technical considerations in ACL reconstruction

ACL reconstruction in the context of quadruple biological restoration presents unique space management challenges. In contrast to the frequent use of bone-block grafts, the anterior tibial tendon allograft represents a particularly suitable alternative in this scenario due to its high biomechanical strength and the absence of additional bone blocks, thereby minimizing the risk of collision with the OCA and the meniscal dovetail [15].

In the femur, the wide arthrotomy allows for direct visualization of the anatomical footprint and independent tunneling. This freedom of orientation is critical to providing a tunnel trajectory that predictably avoids contact or convergence with the osteochondral cylinder implanted in the femoral condyle [15–18]. Anatomical placement centered on the anteromedial (AM) footprint is essential to reduce residual laxity and optimize rotational kinematics [18].

Regarding the tibial technique, it is imperative to adjust the tunnel orientation to approximately 60°–65° in the coronal plane and 50°–55° in the sagittal plane, centered on the native footprint to optimize spatial relationships and preserve bone stock. This specific angulation serves as a surgical space management strategy that allows the graft to pass between the meniscal bone block and the osteochondral cylinder without compromising the structural integrity of the tibial plateau [15–17].

Recent three-dimensional CT modeling studies in combined MAT and ACL reconstruction have quantitatively demonstrated that tunnel/socket overlap most frequently occurs in the subchondral region, particularly between 6 and 10 mm from the tibial articular surface, highlighting this area as the critical zone for convergence risk [24]. These analyses further support that tibial guide inclinations closer to 65° with an anteromedial entry point may reduce tunnel conflict while preserving anatomic footprint positioning [24].

Additional three-dimensional geometric modeling studies in complex concomitant tibial tunnel constructs have demonstrated that unplanned tunnel trajectories on the anteromedial proximal tibia may increase the risk of tunnel convergence, whereas controlled sagittal reorientation and parallel trajectory planning can significantly improve inter-tunnel clearance in simulated conditions [25]. In multiligament settings combining ACL, PCL, and meniscal root tunnels, these studies quantitatively confirmed that convergence risk is not uniformly distributed across all tunnel configurations and may be substantially reduced through trajectory adjustment and spatial planning, reinforcing the broader principle that careful tunnel planning remains critical when multiple biologic reconstructions coexist within the same proximal tibial environment [25]. Likewise, previous cadaveric biomechanical studies have demonstrated that ACL tibial tunnel reaming may directly compromise adjacent meniscal root insertions, producing measurable reductions in root attachment area and biomechanical strength when spatial relationships are not carefully respected [26]. These findings further reinforce the importance of meticulous tunnel planning and adaptive intraoperative trajectory adjustment in complex combined reconstructions [26].

Although these quantitative studies support the biomechanical rationale of trajectory-planning strategies, no formal CT-based validation or cadaveric simulation has been specifically performed for the present four-graft construct. Therefore, the proposed sequential workflow should be interpreted as a technically optimized intraoperative algorithm based on currently available spatial principles, rather than as a quantitatively validated geometric model [24–26].

Finally, while slight modifications in orientation may be required in multi-structural procedures to avoid interference, fundamental anatomical landmarks must not be sacrificed. Furthermore, the perception of increased femoral tunnel verticality on anteroposterior radiographs should not be interpreted as a lack of anatomic positioning, but rather as a consequence of femoral rotation and image intensifier projection. True obliquity is ensured through direct reference to the native footprints and intraoperative multiplanar control [17, 18].

### Final considerations and limitations

The principal limitation of this work is the absence of formal clinical validation within the present manuscript, as the primary objective was to develop and systematize the technical and methodological workflow of combined four-structure biological knee reconstruction rather than to analyze longitudinal clinical outcomes.

Single-stage combined reconstruction offers potential advantages over staged approaches, including reduced surgical exposures, a consolidated rehabilitation process, and early restoration of a biomechanically coherent joint environment. However, this strategy demands strict preoperative planning and precise control of technical interferences between grafts [4, 5, 23].

The sequential workflow described in this review is presented as a structured conceptual framework based on currently available surgical principles and operative experience in complex joint preservation. Formal clinical validation and long-term outcome analysis were beyond the scope of the present technical-methodological review.

Limitations include the high technical demand, a significant learning curve, and the requirement for careful patient selection. This procedure should not be considered an entry-level reconstructive technique. Ideally, surgeons should first acquire independent experience in its major components – including meniscal allograft transplantation, osteochondral allograft transplantation, and complex ligament reconstruction – before attempting a combined four-graft biological reconstruction. A stepwise progression from isolated procedures to combined two- and three-graft reconstructions may represent a reasonable strategy to improve reproducibility and reduce technical complexity.

The availability of suitable allografts and the logistical complexity of simultaneous procurement of multiple size-compatible grafts may represent a major constraint in certain contexts, requiring close coordination with specialized tissue banks and, in some cases, grafts from more than one donor. This requirement may limit the feasibility and reproducibility of the procedure, particularly in regions without established allograft banking infrastructure or advanced tissue procurement systems. Additionally, postoperative rehabilitation is prolonged and requires high patient compliance, limiting its applicability to selected scenarios.

Much of the biological and rehabilitation rationale discussed in this review is necessarily extrapolated from the available literature on isolated osteochondral allograft transplantation, meniscal allograft transplantation, and ligament reconstruction, as direct evidence specifically addressing simultaneous four-graft biological reconstruction remains extremely limited. Therefore, such extrapolations should be interpreted with caution, recognizing that the biological integration, mechanical interactions, and rehabilitation demands of combined multistructural reconstruction may differ substantially from those of isolated procedures.

## Conclusion

The described sequential workflow provides a reproducible methodological framework for the integrated reconstruction of cartilage, meniscus, and ligamentous stability in a single-stage procedure. This approach does not intend to replace established individual techniques but rather offers a structured strategy for their integration in carefully selected complex cases within the paradigm of knee joint preservation.

## Data Availability

Data sharing is not applicable to this article as no new data were created or analyzed in this study.

## Supplementary material

*Supplementary Video 1*: Representative surgical video illustrating key steps of the sequential four-graft biological knee reconstruction workflow, including surgical exposure, recipient femoral shell graft preparation, cadaveric shell graft preparation, dovetail meniscal allograft preparation, final intraoperative stability assessment, and early postoperative knee range of motion. Supplementary material of this article is available at https://www.sicot-j.org/10.1051/sicotj/2026044/olm.

## References

[1] Frank RM, Lee S, Cotter EJ, Hannon CP, Leroux T, Cole BJ (2018) Outcomes of osteochondral allograft transplantation with and without concomitant meniscus allograft transplantation: a comparative matched group analysis. Am J Sports Med 46 (3), 573–580. 29314864 10.1177/0363546517744202

[2] Stone KR, Walgenbach AW, Slatter S, et al. (2025) Meniscus allograft transplantation in conjunction with arthroscopic biologic knee restoration delays arthroplasty in patients older than 50 years. Arthroscopy 41 (4), 1019–1026. 38897483 10.1016/j.arthro.2024.06.008

[3] Powell CW, Norton CD, Colon LF, Wilson AW, Bruce JR (2022) Quadruple hamstring autograft technique for anterior cruciate ligament reconstruction reduces allograft augmentation. Arthrosc Sports Med Rehabil 4 (6), e2059–e2063. 36579051 10.1016/j.asmr.2022.09.006 PMC9791809

[4] Cook JL, Rucinski K, Crecelius CR, Stannard JP (2023) Initial outcomes after unicompartmental tibiofemoral bipolar osteochondral and meniscal allograft transplantation in the knee using MOPS-preserved fresh (viable) tissues. Am J Sports Med 51 (3), 596–604. 36655742 10.1177/03635465221144003

[5] Pawelczyk J, Fanourgiakis I, Feil S, Schneider S, Kougioumtzis I, Siebold R (2025) Clinical results and failure rates after meniscal allograft transplantation and autologous chondrocyte implantation: a systematic review. Knee Surg Relat Res 37, 39. 40983907 10.1186/s43019-025-00291-4 PMC12455755

[6] Meade MJ, Patel K, Sgaglione NA (2023) Current techniques for processing osteochondral allografts are variable: a systematic review. Arthrosc Sports Med Rehabil 5 (2), e123–e131. 10.1016/j.asmr.2025.101151 PMC12447100 40980237

[7] Haikal M, Issac RT, Snow M (2023) Osteochondral allograft transplantation of the knee: a review of indications, techniques, outcome and how to promote biology. Orthop Trauma 37 (3), 161–169.

[8] Firman EJ, Río EM, Sirio AN (2025) Sagittal biplanar “L”-shaped tibial osteotomy associated with osteochondral allograft for symptomatic varus knee and low patella. Int J Surg Case Rep 137, 112074. 41541129 10.1016/j.ijscr.2025.112074 PMC12621443

[9] Dwyer T, Ryan MC, Rita K, et al. (2017) Reliability and validity of the arthroscopic International Cartilage Repair Society classification system: correlation with histological assessment of depth. Arthroscopy 33 (6), 1211–1217. 28162918 10.1016/j.arthro.2016.12.012

[10] Tang D, Song H, Yan C, Luo Y, Su X, Ruan S (2025) Hydrogel composite scaffold repairs knee cartilage defects: a systematic review. RSC Adv 15 (13), 10337–10364. 40200956 10.1039/d5ra01031d PMC11976716

[11] Kester CR, Caldwell PE 3rd, Pearson SE (2021) Lateral meniscal allograft transplant: dovetail bone bridge preparation. Arthrosc Tech 10 (4), e969–e973. 33981538 10.1016/j.eats.2020.11.008 PMC8084757

[12] Worhacz KM, Carter TR (2023) Meniscal allograft transplantation: does surgical technique influence clinical outcomes? Curr Rev Musculoskelet Med 16 (5), 163–172. 37014608 10.1007/s12178-023-09825-3 PMC10188795

[13] Wang T, Bugbee WD (2025) Osteochondral allograft transplantation. Clin Sports Med, 44 (3), 553–564. 40514155 10.1016/j.csm.2024.12.002

[14] Dave U, Villarreal-Espinosa JB, Shah H, Cotter EJ, Gómez-Verdejo F, Carpenter M, Gerhold C, Mamonov A, Chahla J, Verma NN (2025) Patients have similar clinical outcomes and failure rates after anterior cruciate ligament reconstruction with tibialis anterior tendon, bone-patellar tendon-bone, hamstring tendon, or Achilles tendon allografts: a systematic review. Arthrosc Sports Med Rehabil 7 (2), 101035. 40297074 10.1016/j.asmr.2024.101035 PMC12034080

[15] Dadoo S, Herman ZJ, Hughes JD (2024) Surgical techniques in primary ACL reconstruction: getting it right the first time. Clin Sports Med 43 (3), 399–412. 38811118 10.1016/j.csm.2023.08.007

[16] Shankar DS, Vasavada KD, Avila A, DeClouette B, Aziz H, Strauss EJ, Alaia MJ, Jazrawi LM, Gonzalez-Lomas G, Campbell KA (2023) Acceptable clinical outcomes despite high reoperation rate at minimum 12-month follow-up after concomitant arthroscopically assisted anterior cruciate ligament reconstruction and medial meniscal allograft transplantation. Knee Surg Relat Res 35 (1), 2. 36627709 10.1186/s43019-023-00176-4 PMC9832613

[17] Mhaskar VA, Jain Y, Soni P, Fiske R, Maheshwari J (2021) How important is the tunnel position in outcomes post-ACL reconstruction: a 3D CT-based study. Indian J Orthop 56 (2), 312–318. 35140863 10.1007/s43465-021-00485-4 PMC8789976

[18] Kim SJ, Song SY, Kim TS, Kim YS, Jang SW, Seo YJ (2021) Creating a femoral tunnel aperture at the anteromedial footprint versus the central footprint in ACL reconstruction: comparison of contact stress patterns. Orthop J Sports Med 9 (4), 23259671211001802. 33997070 10.1177/23259671211001802 PMC8111278

[19] Firman EJ, Sirio AN, Río EM (2025) Injuria arterial severa asociada a la obtención de injertos isquiotibiales autólogos en la reconstrucción artroscópica de ligamento cruzado anterior. Rev Latinoam Artrosc Reconstr Artic Trauma Deport (RELART) 32 (2), 169–176.

[20] Cook JL, Rucinski K, Crecelius CR, Kfuri M, Stannard JP (2024) Treatment failures (revision or arthroplasty) after knee osteochondral allograft transplantation with minimum two-year follow-up. Knee 46, 128–135. 38128151 10.1016/j.knee.2023.12.002

[21] Lydon KL, Struijk C, Michielsen J, Prokop L, Krych AJ, Saris D, Verdonk P (2024) Fresh versus frozen meniscal allograft transplant: revisit or redundant? A systematic review. Am J Sports Med 52 (8), 2159–2167. 38282584 10.1177/03635465231200236

[22] Runer A, Keeling L, Wagala N, Nugraha H, Özbek EA, Hughes JD, Musahl V (2023) Current trends in graft choice for primary anterior cruciate ligament reconstruction – part II: In-vivo kinematics, patient reported outcomes, re-rupture rates, strength recovery, return to sports and complications. J Exp Orthop 10 (1), 40. 37014518 10.1186/s40634-023-00601-3 PMC10073382

[23] Bi AS, Franzia CH, Mufti YN, Sachs JP, Cole BJ (2025) Short-term patient-reported outcomes after osteochondral allograft combined with concomitant knee procedures predict mid- and long-term outcomes. Arthroscopy 41 (10), 4168–4176. 40209834 10.1016/j.arthro.2025.03.063

[24] DeFroda SF, Albuquerque JBI, Bezold W, et al. (2024) Optimizing socket-tunnel position for meniscal allograft transplantation combined with ACL reconstruction: a 3D model analysis. Orthop J Sports Med 12 (6), 23259671241246277. 38845611 10.1177/23259671241246277 PMC11155341

[25] Gursoy S, Perry AK, Brady A, et al. (2022) Optimal tibial tunnel placement for medial and lateral meniscus root repair on the anteromedial tibia in the setting of anterior and posterior cruciate ligament reconstruction of the knee. Am J Sports Med 50 (5), 1237–1245. 35225000 10.1177/03635465221074312

[26] LaPrade CM, Smith SD, Rasmussen MT, Hamming MG, Wijdicks CA, Engebretsen L, Feagin JA, LaPrade RF (2015) Consequences of tibial tunnel reaming on the meniscal roots during cruciate ligament reconstruction in a cadaveric model, part 1: the anterior cruciate ligament. Am J Sports Med 43 (1), 200–206. 25361859 10.1177/0363546514554769

